# Preoperative chemoradiotherapy for rectal cancer: the sensitizer role of the association between miR-375 and c-Myc

**DOI:** 10.18632/oncotarget.19393

**Published:** 2017-07-19

**Authors:** Raquel Conde-Muiño, Carlos Cano, Victoria Sánchez-Martín, Antonio Herrera, Ana Comino, Pedro P. Medina, Pablo Palma, Marta Cuadros

**Affiliations:** ^1^ Division of Colon & Rectal Surgery, University Hospital Virgen de las Nieves, Granada, Spain; ^2^ Department of Computer Science and Artificial Intelligence, University of Granada, Granada, Spain; ^3^ Department of Biochemistry, Molecular Biology III and Immunology, University of Granada, Granada, Spain; ^4^ GENYO, Centre for Genomics and Oncological Research, Pfizer/University of de Granada/Junta de Andalucía, PTS Granada, Granada, Spain; ^5^ Department of Biochemistry and Molecular Biology I, University of Granada, Granada, Spain

**Keywords:** regulatory networks, chemoradiotherapy-resistance, rectal cancer, miRNAs, c-Myc

## Abstract

Administration of chemoradiation before tumor resection has revolutionized the management of locally advanced rectal cancer, but many patients have proven resistant to this preoperative therapy. Our group recently reported a negative correlation between c-Myc gene expression and this resistance. In the present study, integrated analysis of miRNA and mRNA expression profiles was conducted in 45 pre-treatment rectal tumors in order to analyze the expressions of miRNAs and c-Myc and their relationship with clinicopathological factors and patient survival. Twelve miRNAs were found to be differentially expressed by responders and non-responders to the chemoradiation. Functional classification revealed an association between the differentially expressed miRNAs and c-Myc. Quantitative real-time PCR results showed that miRNA-148 and miRNA-375 levels were both significantly lower in responders than in non-responders. Notably, a significant negative correlation was found between miRNA-375 expression and c-Myc expression. According to these findings, miRNA-375 and its targeted c-Myc may be useful as a predictive biomarker of the response to neoadjuvant treatment in patients with locally advanced rectal cancer.

## INTRODUCTION

Standard treatment of locally advanced rectal cancer (LARC) includes 5-fluorouracil (5-FU) based neoadjuvant chemoradiotherapy (CT/RT) followed by surgery [1]. This CT/RT regimen reduces the local recurrence rate, and a good response to this treatment improves the overall prognosis [2]. However, the clinical effectiveness of 5-FU is limited by drug resistance [3], and novel strategies are required to increase its therapeutic effect and to determine the molecular mechanisms underlying the therapeutic response. This may enable the identification of patients who would respond to this treatment.

A study by our group using microarray analysis and quantitative real time PCR (qRT-PCR) found that higher c-Myc mRNA expression, in the absence of amplification, was associated with an improved response to preoperative 5-FU-based CT/RT in LARC [4]. Overexpression of the c-Myc transcription factor is frequently observed in various types of cancer and plays a major role in the regulation of apoptosis, proliferation, and/or self-renewal, among other cellular activities, and in the resistance to drugs [5]. However, the mechanisms by which c-Myc expression levels influence the response of LARC patients to neoadjuvant chemoradiation are poorly understood. Various mechanisms can affect mRNA expression levels. For instance, dysregulation of microRNA (miRNA) expression, a post-transcriptional mechanism, may lead to gene expression changes in cancer. Thus, miRNAs act as negative regulators for mRNA expression via sequence-complementary targeting of 3′-untranslated regions (3′-UTRs) and other regions in order to repress translation or mediation of mRNA [6, 7]. Because their target genes can control multiple biological processes, including apoptosis, proliferation, and differentiation, some miRNAs have been characterized as tumor suppressor genes or proto-oncogenes [8]. There is increasing evidence of significant differences in miRNAs in colorectal cancer that may be of potential clinical value for the diagnosis [9], treatment [10] and prognosis [11]. MiRNA expression in rectal tumors is less well documented, although recent studies have reported the differential expression of several miRNAs related to the therapeutic response and prognosis [12–22]. However, there is a very small overlap among reported sets of dysregulated markers, with only miR-21 [13, 16, 19] and miR-630 [15, 20] being described in common across published molecular signatures.

In the present study, integrative analysis of miRNA and gene expression profiles was conducted in LARC patients to detect c-Myc-targeted miRNAs that are also associated with the response to neoadjuvant treatment. Twelve miRNAs were identified, and a regulatory network based on the expressions of these miRNAs and c-Myc was created. Among the 12 miRNAs, application of the miRWalk database showed miRNA-target interactions with c-Myc for miR-145, miR-148, miR-375 and let-7f, finding miR-375 to be the most promising biomarker of the therapeutic response. These findings contribute to a better understanding of the molecular mechanisms underlying regulation of the response to neoadjuvant treatment in rectal cancer.

## RESULTS

### Patient and tumor characteristics

The study included 45 patients with LARC (15 responders and 30 non-responders to neoadjuvant chemoradiation). Data on their age, sex, disease stage, response to chemoradiation, surgical technique, and overall survival are reported in Supplementary Table 1 (Supplementary Material). No statistically significant differences in age, sex, disease stage, or surgical technique were observed between responders and non-responders (Supplementary Table 2, Supplementary Material).

### Comparison of miRNA expression patterns between responders and non-responders

A total of 12 miRNAs were dysregulated in responders but not in non-responders (p <0.05; non-paired t-test), with 11 being downregulated (miR-30b, miR-145, miR-148a, miR-375, miR-451, miR-519b-3p, miR-650, miR-1183, miR-1233, miR-1243, and let-7f) and one upregulated (miR-18a). The dysregulated miRNAs all showed a fold change of > 2, with miR-1243 being downregulated almost 10-fold, let-7f 250-fold and miR-519b-3p 1000-fold. Ten of these miRNAs (miR-18a, miR-30b, miR-148a, miR-375, miR-451, miR-519b-3p, miR-650, miR-1233, miR-1243, and let-7f) have never previously been associated with the response to treatment in rectal adenocarcinomas.

### Regulatory network of miRNAs and target genes

**Figure 1:**
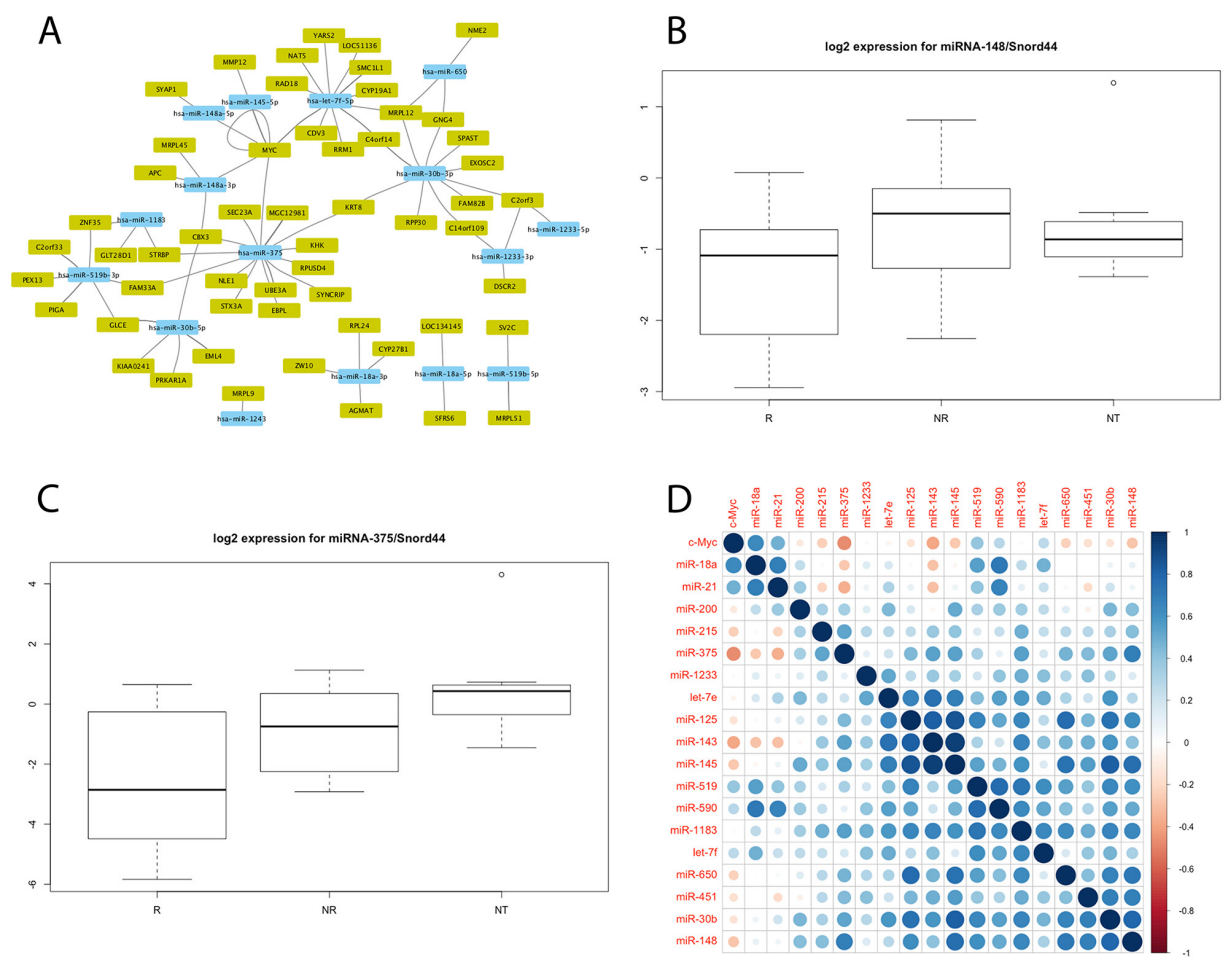
**(A)** Cytoscape regulatory network of the 94 mRNA-miRNA pairs differentially expressed (p<0.05) by responders (R) and non-responders (NR); **(B, C)** Expressions of miR-148 (B) and miR-375 (C) assessed by individual TaqMan assays in LARC and in normal adjacent rectal tissue. Box plots represent percentiles 25 and 75 of expression for each group of patients, and the ends of the vertical lines represent maximum and minimum expressions. For each miRNA, statistically significant differences were found between responders and non-responders. miR-375 was also downregulated in LARC compared with normal adjacent tissues (NT). There is no significant difference in miR-148 levels between the tumor subgroup and normal rectal tissue subgroup (p=0.33); **(D)** Pearson’s correlation plot for miRNA against c-Myc expression profiles determined by Taqman individual assays. Pearson’s correlation values computed for each miRNA and c-Myc expression values are represented as circles whose sizes are proportional to the absolute correlation value, with colors ranging from dark red (coefficient -1), to dark blue (coefficient 1), as depicted in the color scale. The figure was generated with the ‘corrplot’ library in R.

Interactions in our two array datasets between the mRNAs and miRNAs related to the therapeutic response were validated using miRWalk. We combined the list of 257 mRNAs previously published by our group [4] with the 12 miRNAs identified by TaqMan® OpenArray® MicroRNA Panels. Ninety-four potential miRNA-target gene pairs were identified, and their network representation showed c-Myc to be the target gene that interacted with the largest number of miRNAs, including miR-145, miR-148, miR-375, and let-7f (Figure 1A).

### Evaluation of the potential of c-Myc-targeted miRNAs as biomarkers of the therapeutic response

We investigated the c-Myc sequence and the structural features of miRNAs found in the databases consulted and the literature, identifying 82 miRNAs that target c-Myc mRNA (Supplementary Table 3, Supplementary Material). After excluding the miRNAs whose interactions with c-Myc were supported by only one item of evidence, either from the databases or the literature [12–16, 18–22], 29 candidates remained. Six of these (miR-21.5p, miR-125, miR-143.3p, miR-200c, miR-215.5p, and let7e) and 12 miRNAs identified by TaqMan microRNAarray (miR-18a, miR-30b, miR-145, miR-148a, miR-375, miR-451, miR-519b-3p, miR-650, miR-1183, miR-1233, miR-1243, and let-7f) were included in the candidate list for qRT-PCR validation using TaqMan® Gene Expression Assays. A good correlation was observed between the microarray analysis and qRT-PCR results. Out of the miRNAs selected for qRT-PCR validation, the expressions of miR-148 (p=0.032) and miR-375 (p=0.011) significantly differed between responders and non-responders to the neoadjuvant chemotherapy (Figure 1B and 1C), being markedly downregulated in the responders, especially in the case of miR-375. The area under the ROC curve (AUC) value for the accuracy of miR-375 to differentiate between responders and non-responders was 0.644 (95% CI: 0.447–0.842; 0.60 for sensitivity and 0.61 for specificity). A lower AUC value of 0.581 (95% CI: 0.384–0.779, 0.60 for sensitivity and 0.61 for specificity) was found for miR-148. In comparison to these values, the combination of miR-375 and miR-148 showed lower sensitivity (0.42) and higher specificity (0.72). Because miR-148 and miR-375 levels were lower in responders than in non-responders, their levels in normal adjacent rectal tissue samples (N= 8) was investigated. As shown in Figure 1B, there was no change in miR-148 levels between the tumor and normal rectal tissue groups (p=0.33), whereas the expression of miR-375 was lower in the tumor samples (p=0.006), supporting the proposal that miR-375 may act as a tumor suppressor gene in rectal cancer (Figure 1C).

Finally, significant differences in c-Myc expression were found between responders and non-responders (p=0.024). c-Myc expression also differed between the tumor and normal adjacent tissue groups, being higher in the former (p<0.001) (Supplementary Figure 1, Supplementary Material). These results confirm that c-Myc mRNA is a potentially valuable biomarker of the response to treatment in LARC.

### Correlations between miRNA and c-Myc mRNA levels in LARC patients

Pearson’s correlation coefficients were determined for the 18 miRNAs-c-Myc pairs validated by Taqman individual assays (Figure 1D). Ten of these miRNAs showed a negative Pearson’s correlation coefficient with c-Myc gene (miR-30b, miR-125, miR-143, miR-145, miR-148, miR-200, miR-215, miR-375, miR-451, and miR-650) and five of them (miR-18a, miR-21, miR-519, miR-590, and let7f) a positive correlation, while three other miRNAs (miR-1183, miR-1233, and let7e) showed almost no correlation. The highest negative correlation coefficient was found between c-Myc and miR-375 (Pearson’s correlation=-0.467, p<0.001), followed by miRNA-148 (Pearson’s correlation=-0.278, p=0.037).

### Association between c-Myc and miRNAs and survival

Mean mRNA expressions of c-Myc, miR-148, and miR-375 were studied by Cox regression analysis to establish their association with patient survival, classifying the expression of samples as high or low according to TaqMan® Gene Expression Assay data. In the rectal tumors studied, no significant association was found between c-Myc, miR-148, or miR-375 expression levels and overall survival (OS). Thus, the mean OS was 87 months in the low c-Myc expression subgroup and 83 months in the high expression subgroup (p=0.971). Although all patients in the low miR-148 expression subgroup were alive at the end of the study (mean follow up of 83 months), the difference between subgroups did not reach statistical significance (p=0.133), probably attributable to the small sample size. Likewise, despite the improved OS observed in the low versus high miR-375 expression subgroups (91.7 months versus 86 months, p=0.993), the difference was not statistically significant. Since all patients were treated with equivalent chemotherapy protocols, it is not likely that differences in treatment can explain variations in survival.

### Influence of c-Myc expression on susceptibility of cancer cells to 5-FU

The susceptibility of cancer cells to 5-FU was tested in three cell lines: two that have demonstrated medium (SW480) and high (SW837) resistance to CT/RT in colorectal cancer [23] and show the typical chromosomal and transcriptional aberrations observed in primary colorectal cancers; and a metastatic colorectal cancer cell line (SW620) derived from the same patient as SW480. The MTT assay was applied after 48 or 72 h of continuous drug incubation, recording the LC50 (50% of untreated control cell count). The LC50 values markedly differed between SW837 and the other two cell lines (Supplementary Table 4, Supplementary Material), with values at 72 h of incubation of 1.16 μM and 1.15 μM for SW480 and SW620 *versus* 80 μM for SW837, more than 69-fold higher. The possibility that c-Myc may mediate resistance to 5-FU was explored by measuring the mRNA expression and protein level of c-Myc in these tumor cell lines by qRT-PCR and Western blot, respectively, finding them to be positively correlated with the response to 5-FU (Supplementary Figure 2, Supplementary Material). The lowest c-Myc mRNA expression and protein levels were observed in the strongly 5-FU-resistant SW837 cell line and the highest in the more susceptible SW480 and SW620 cell lines.

## DISCUSSION

Based on recommendations of the German Rectal Cancer Study Group, the standard approach to LARC is neoadjuvant CT/RT, with the concurrent addition of a systemic cytotoxic agent during the 5-week course of radiation. However, questions have been raised about this approach, given that a pathological complete response is reported in only a minority of patients. Scientists have also debated the reason for the improved systemic control of the disease achieved by the combined treatment, besides its known local effect. Because radiation has the capacity to exert convincing biological effects in a given tumor volume, it has been argued that the improvement in systemic outcomes is due to the increased likelihood of eliminating the source responsible for maintaining the population of tumor cells [24].

There is a need for predictive biomarkers to assess the response to neoadjuvant CT/RT and to support the stratification of patient risk for treatment personalization. In this regard, several studies have reported the potential usefulness of mRNA and miRNA expression signatures [20, 21, 25] to predict the pathological response in rectal cancer patients. However, the small overlap between rectal cancer gene sets has led to questions about their biological significance*.* This study of miRNA expression profiles in 22 pre-treatment biopsies from LARCs identified 12 miRNAs that were differentially expressed by responders and non-responders to pre-surgical FU-based CT/RT, 10 of which (miR-18a, miR-30b, miR-148a, miR-375, miR-451, miR-519b-3p, miR-650, miR-1233, miR-1243, and let-7f) have not previously been associated with rectal cancer. The expression of all differentially expressed miRNAs, with the exception of miR-18a, was significantly lower in responders than in non-responders. Results of qRT-PCR analysis also revealed differences between responders and non-responders, but significance was only reached for miR-148 and miR-375. This is the first report that miR-148 and miR-375 are associated with the response to CT/RT. In addition, miR-375 was significantly downregulated in pretreatment tumor biopsy samples than in normal tissue samples adjacent to the tumor. Although downregulation of miR-375 has been reported in human colorectal cancer [26, 27] and described as useful for cancer subtyping [28–30], it has not previously been recorded in rectal cancer. The present findings that miR-148 and miR-375 are potentially useful as biomarkers of the response to 5-FU-based CT/RT. ROC curve analysis confirmed that responders can be differentiated from non-responders based on their miR-375 expression (AUC value = 0.644). The expression of miR-375 also serves as a biomarker of the presence of rectal cancer.

Previous studies of cell lines and organisms reported that miRNAs downregulate steady-state levels of their target mRNAs [7]. In the present investigation, mRNA profiles available from the same LARC patients [4] were used to identify the miRNAs that interact with these mRNAs. The gene most frequently involved in the 94 miRNA - mRNA interactions observed was the c-Myc gene, and the results of qRT-PCR and Pearson’s correlation analysis confirmed the miRNA-c-Myc pairs identified. The negative correlation found between miR-375 and c-Myc mRNA expression was previously observed in a functional study of oral cancer [31], and the authors concluded that miR-375 indirectly affects c-Myc expression by regulating expression of the Cancerous Inhibitor of PP2A (CIP2A), which stabilizes c-Myc and prevents its degradation. A previous study by our group in LARC patients reported overexpression of c-Myc mRNA in responders in the absence of c-Myc amplification, suggesting that c-Myc activation does not depend on gene amplification in rectal cancer [4]. Based on these data and the present findings, we propose that miR-375 downregulation may be a mechanism underlying the overexpression of c-Myc in LARC. MiR-375 may act as a tumor suppressor by targeting CIP2A, and its expression was lower in tumor than in normal rectal tissues, especially among the responders, suggesting downregulation of the miR-375 pathway in these tumors. No statistically significant differences in OS were found as a function of c-Myc, miR-148, or miR-375 levels, although all patients with low miR-148 expression were alive at the end of the study (mean follow-up of 83 months). Some miRNAs have been related to survival in rectal cancer [32], but the prognostic value of miRNAs in this disease requires further investigation [33, 34]. MiRNA expression patterns were reported to significantly differ between colon and rectal cancer, probably due to differences in genetic abnormalities and treatment protocols [35].

Although 5-FU chemotherapy is a well-established adjuvant treatment of colorectal cancer (CRC), many patients acquire resistance during therapy [36]. Overexpression of c-Myc has been identified in rectal cancer, but the role of c-Myc in the response to 5-FU remains unclear [4]. Better knowledge of c-Myc function could increase our understanding of the biology of the responder rectal cancer patients but may also provide a novel therapeutic molecular target for clinical practice. To test whether c-Myc contributes to CT/RT-resistance, we tested the response to 5-FU in three tumor cell lines with different c-Myc expression levels. The expression of c-Myc was positively correlated with 5-FU resistance in the present study, confirming previous findings on the association between c-Myc and the response to pre-surgical CT/RT in patients with LARC [4]. These results support the proposal that c-Myc enhances the susceptibility to 5-FU of colorectal cancer cells by promoting cell cycle S-phase entry and therefore represents a novel therapeutic target in colorectal cancer treatment. In brief, the downregulation of the tumor suppressor miR-375 observed in responders would abolish the post-transcriptional regulation of c-Myc, contributing to its protection from degradation. The resulting increase in c-Myc expression would facilitate incorporation of 5-FU into the S phase of the cell cycle, increasing cell susceptibility to this compound [4]. According to these data, c-Myc may be an attractive therapeutic target in patients with LARC or other miR-375 deficient tumors.

In conclusion, we identified 12 dysregulated miRNAs in rectal tumors, many of which had not previously been reported. Expressions of miR-148 and miR-375 were downregulated in samples from patients with LARC and may also be useful biomarkers of the response to neoadjuvant chemoradiation.

## MATERIALS AND METHODS

### Patient samples

The study included from University Hospital Virgen de las Nieves patients with LARC treated at our tertiary level referral hospital between 2008 and 2017.

Inclusion criteria were: histologically confirmed rectal tumor at clinical stage UICC II-III (cT3-4/and or N positive) on endorectal ultrasound and/or MRI scan. Exclusion criteria were: tumor localized at > 13 cm from the anal verge (by rigid rectoscopy); the presence of synchronic colonic cancer (by colonoscopy) or distant metastases (by 18FDG PET-CT), or suspicion of hereditary colorectal cancer.

All patients included signed their informed consent to participation in the study, which was approved by the Ethics Committee of Granada Province (Spain).

Before treatment started, a biopsy of the tumor was done in all 45 patients, and samples of histologically cancer-free adjacent tissue from 8 of these were also prospectively collected and freshly frozen. Patients then received a total dose of 50.4Gy of radiation (28 fractions of 1.8Gy) associated with capecitabine (oral form of 5-FU) with or without oxaliplatin, according to our hospital protocol. At 8-10 weeks after completion of their CT/RT treatment, all patients underwent standardized surgery, including total mesorectal excision.

The therapeutic response was assessed in surgical specimens using the semi-quantitative tumor regression grading (TRG) system of Mandard, classifying patients with scores of TRG1 or TRG2 as responders and those with scores of TRG3, TRG4, or TRG5 as non-responders [37].

### RNA extraction

Total RNA was extracted from 10-20 mg of the 45 rectal tumor samples after their homogenization in TRIzol Reagent using a TissueLyser (Qiagen, Valencia, CA), applying the TRIzol protocol. RNA quality was evaluated with an Agilent 2100 Bioanalyzer.

### MiRNA expression profiling

Twenty-two samples (10 from responders and 12 from non-responders) were randomly selected for miRNA profiling with TaqMan® OpenArray® MicroRNA Panels (Life Technologies); these consist of DNA probes representing 754 human miRNAs from the Sanger miRBase (Version 14), synthesized *in situ* using the one-color technique according to the manufacturer’s instructions. Thermo Fisher Cloud Software, version 1.0 (Life Technologies Corp., CA, USA) was used for data normalization and analysis. Global normalization was applied to normalize the qPCR data [38]. The miRNA data are available through GEO accession number GSE98959. Probes with ROX signal values under 2000 were filtered out, and the maximum cut-off Crt value was 26 (setting all probes with higher values at 26). An unpaired Student’s t-test was then performed to identify probes that significantly differed (p<0.05) between responders and non-responders.

### mRNA signature for response to treatment

We previously reported the gene expression profiles of 26 pre-treatment biopsies from LARC patients without metastases (10 responders and 16 non-responders) using the Human WG CodeLink microarray platform^4^, finding that 257 genes were differentially over-expressed in responders. Raw and normalized gene expression values for each sample in the study are publically available at the Gene Expression Omnibus GEO database (submission n° GSE5378).

### Combination of miRNA and mRNA gene expression profiling

Target genes of the differentially expressed miRNAs were predicted by using six bioinformatic algorithms (DIANAmT, miRanda, miRDB, miRWalk, PICTAR and Targetscan) included in miRWalk. Data were downloaded on 2 August 2016.

### In silico analysis of miRNAs for c-Myc gene

The list of potential miRNAs for c-Myc was based on data from the microRNA, mirTArBase, picTar, miRCancer, Targetscan, UCSC Genome browser, microRNA.org, and miRSearch 3.0 (Exiqon) databases. Data were downloaded on 25 January 2016.

### Quantitative RT-PCR analysis

In order to validate our results, qRT-PCR analysis of c-Myc mRNA was performed in the 12 miRNAs identified by TaqMan® OpenArray® MicroRNA Panels and in 6 representative miRNAs selected from the databases and literature. Assays-on-Demand Taqman probes (ThermoFisher) of c-Myc mRNA and microRNAs were used (Supplementary Material Supplementary Table 5), and PCR was conducted with the ABI prism 7900 system (Applied Biosystems) following the manufacturer’s recommendations. Relative quantification of the expression of the genes was performed by constructing a standard curve with at least four different concentrations (in triplicate). Stably expressed endogenous controls Gapdh and Snord44 were selected based on expression data (standard deviation lower than 0.8 across all analyzed samples). All experiments were performed in triplicate, and the mean value was used. Relative expressions of c-Myc and each miRNA were calculated by the comparative Ct method, normalizing the data using the mean Ct value of the selected endogenous control. Normalized data were log^2^ transformed. Differences in gene expression between responder and non-responder groups were evaluated with the Student’s t-test.

### Cell cultures

SW480, SW620, and SW837 cell lines from the American Type Culture Collection (ATCC) were cultured at 37 °C with 5 % CO_2_ atmosphere in RPMI medium supplemented with 10 % fetal bovine serum, penicillin, streptomycin and amphotericin, as recommended by the ATCC. Cell lines were characterized according to the literature and our c-Myc genotyping results (Supplementary Table 6, Supplementary Material).

### Determination of LC50 of 5-FU

Drug sensitivity was determined by MTT (3-(4,5-Dimethylthiazol-2-yl)-2,5-diphenyltetrazolium bromide) after continuous drug incubation for 48 h or 72 h, using 5-FU concentrations ranging between 160 μM and 0.3nM to estimate LC50 values. Cells were seeded at 1x10^4^ cells/well in 200 μl culture medium and incubated at 37 °C in 5 % CO_2_. After 24 h, the medium was replaced with a final volume of 200 μl medium plus 5 μl compound (dilution 1/40) and controls were added to the plates, using 8 mM methyl methanesulfonate (MMS) as positive control and 0.5% DMSO as negative control, after a preliminary study found that 1% DMSO had no effect on these cell lines. A doxorubicin curve was used as internal control. When compound and controls were added, plates were incubated at 37°C in 5 % CO_2_ for 48 or 72 h.

The MTT solution was prepared at 5 mg/ml PBS and diluted at 0.5 mg/ml in MEM without phenol red. For the MTT assay, the sample solution in each well was flicked off and 100 μl MTT dye was added, followed by gently shaking of the plates and their incubation for 3 h at 37 °C in 5 % CO_2_. After removal of the supernatant, 100 μl 100 % DMSO were added, and the plates were gently shaken to solubilize the formazan formed, measuring the absorbance at a wavelength of 570 mM.

### Western blot

Total protein was extracted using RIPA lysis buffer containing 1 % PMSF and 1 % protease inhibitor cocktail [39]. Lysates were subjected to Western blot analysis to determine c-Myc protein levels (Cell Signalling Technology, cat. no 5605), using actin (Sigma, cat. no A5441) as primary antibody and following the manufacturer’s instructions.

### Statistical analysis

Differences in clinicopathological characteristics between responders and non-responders were analyzed using the Student’s t-test for continuous variables, and the chi-square or two-sided Fisher exact test for categorical variables. The unequal variances t-test (Welch) was applied to determine the statistical significance of differences in transcript levels. Receiver operating characteristic (ROC) curves were constructed to establish cutoff values for miRNAs and c-Myc expressions that provide optimal specificity and sensitivity to differentiate between responders and non-responders. After classification of the patients according to these cutoff values, Kaplan-Meier curves were constructed to evaluate the OS using the Log-Rank test. SPSS version 15.0 (SPSS Inc., Chicago, IL) and R version 3.3.0 were used for the data analyses. The sample size for the miRNA microarray experiments was calculated to obtain a power of 0.8.

## SUPPLEMENTARY MATERIALS FIGURES AND TABLES 








