# Correction: Preoperative chemoradiotherapy for rectal cancer: the sensitizer role of the association between miR-375 and c-Myc

**DOI:** 10.18632/oncotarget.27541

**Published:** 2020-03-31

**Authors:** Raquel Conde-Muiño, Carlos Cano, Victoria Sánchez-Martín, Antonio Herrera-Merchan, Ana Comino, Pedro P. Medina, Pablo Palma, Marta Cuadros

**Affiliations:** ^1^ Division of Colon & Rectal Surgery, University Hospital Virgen de las Nieves, Granada, Spain; ^2^ Department of Computer Science and Artificial Intelligence, University of Granada, Granada, Spain; ^3^ Department of Biochemistry, Molecular Biology III and Immunology, University of Granada, Granada, Spain; ^4^ GENYO, Centre for Genomics and Oncological Research, Pfizer/University of de Granada/Junta de Andalucía, PTS Granada, Granada, Spain; ^5^ Department of Biochemistry and Molecular Biology I, University of Granada, Granada, Spain

**This article has been corrected:** The name of the 4th author in the listing has been updated, correcting the name as follows:

**Antonio Herrera-Merchan^4,5^**

Original article: Oncotarget. 2017; 8:82294–82302. 82294-82302. https://doi.org/10.18632/oncotarget.19393

