# Supplementary material for: Preoperative chemoradiotherapy for rectal cancer: the sensitizer role of the association between miR-375 and c-Myc
Source: Oncotarget. 2017 Jul 19;8(47):82294–302. doi: 10.18632/oncotarget.19393 (PMC5669890; doi:10.18632/oncotarget.19393)
Supplement: Supplementary file 1 [file oncotarget-08-82294-s001.pdf]

Preoperative chemoradiotherapy for rectal cancer: the sensitizer role of the association between miR-375 and c-Myc

SUPPLEMENTARY MATERIALS

Supplementary Table 1: Characteristics of the patients with rectal tumor.

See Supplementary File 1

Supplementary Table 2: Patient characteristics stratified by response to treatment.

See Supplementary File 2

Supplementary Table 3: 82 miRNAs manually annotated in database as related to c-Myc gene and/or identified in the literature. 29 candidate miRNAs described in more than one database or article are highlighted in grey.

See Supplementary File 3

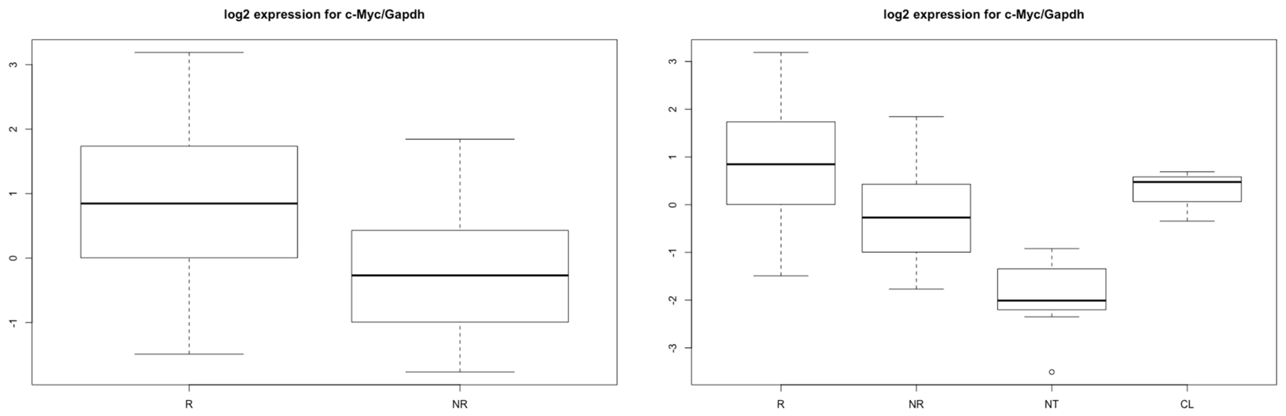

Supplementary Figure 1: c-Myc mRNA is a potential biomarker of response to treatment in LARC. Box plots representing expression values of gene c-Myc by real-time RT-PCR in responders (R) and non-responders (NR). Boxes represent quartiles, the median is represented by a black line within the box, and circles represent atypical values (1.5- to 3-foldthe length of the box). Significant differences in the expression of c-Myc were found among the subgroups. NT: non-tumor samples; CL: cell lines (SW480, SW620 and SW837).

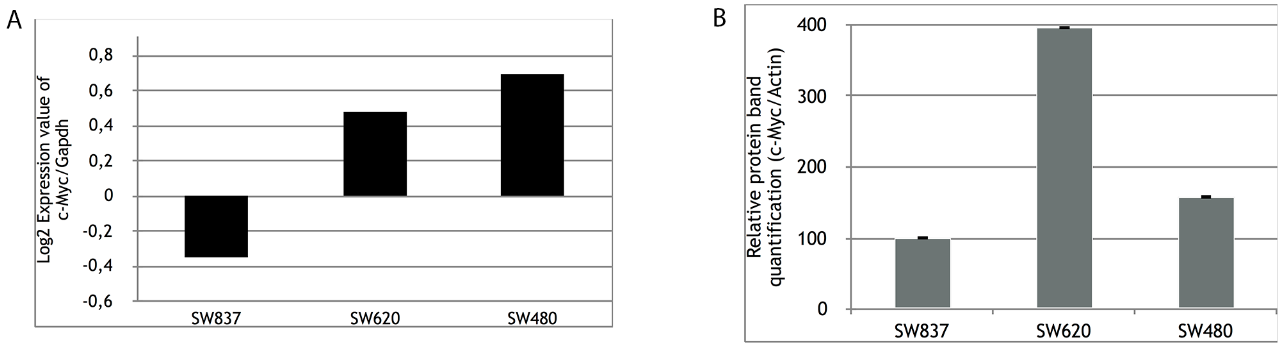

Supplementary Figure 2: c-Myc mRNA and protein expression levels in SW480, SW620, and SW837. (A) TaqMan mRNA assay for c-Myc. c-Myc expression was normalized using Gapdh expression levels. SW837 cell line expressed the lowest c-Myc levels; **(B)** c-Myc protein by Western blot. Relative protein band quantification (c-Myc/Actin) was performed by optical density scanning.

Supplementary Table 4: Determination of lethal concentration (LC50) of 5-FU in S480, SW620, and SW837 cell lines

	MTT 48 h	MTT 72 h
SW480	0.95 µM	1.16 µM
SW620	0.76 µM	1.15 µM
SW837	>160 µM	80 µM

Cells were incubated with the drug for 48 h or 72 h. Viability was determined by a MTT assay to detect metabolic activity. Each sample at each concentration was run in quadruplicate and was normalized to control samples.

Supplementary Table 5: Taqman probes and primers used for the qRT-PCR analysis

	Target	Sequence	Assay ID Thermo Fisher Scientific
**mRNA**	c-Myc		Hs00153408_m1
	Gapdh		Hs02758991_g1
		**miRNA database results**	
**miRNA**	hsa-miR-21-5p	UAGCUUAUCAGACUGAUGUUGA	477975_mir
	hsa-miR-125	UCCCUGAGACCCUAACUUGUGA	477885_mir
	hsa-miR-143-3p	UGAGAUGAAGCACUGUAGCUC	477912_mir
	hsa-miR-200c-3P	UAAUACUGCCGGGUAAUGAUGGA	478351_mir
	hsa-miR-215-5p	AUGACCUAUGAAUUGACAGAC	478516_mir
	let7c-5e	UGAGGUAGGAGGUUGUAUAGUU	478579_mir
		**miRNA array results**	
**miRNA**	miR-18a	UAAGGUGCAUCUAGUGCAGAUAG	478551_mir
	miR-30b	UGUAAACAUCCUACACUCAGCU	478007_mir
	miR-145	GUCCAGUUUUCCCAGGAAUCCCU	477916_mir
	miR-148a	UCAGUGCACUACAGAACUUUGU	477814_mir
	miR-375	UUUGUUCGUUCGGCUCGCGUGA	478074_mir
	miR-451	AAACCGUUACCAUUACUGAGUU	478107_mir
	miR-519b-3p	AAAGUGCAUCCUUUUAGAGGUU	479333_mir
	miR-650	AGGAGGCAGCGCUCUCAGGAC	479129_mir
	miR-1183	CACUGUAGGUGAUGGUGAGAGUGGGCA	477870_mir
	miR-1233	UGAGCCCUGUCCUCCCGCAG	477877_mir
	miR-1243	AACUGGAUCAAUUAUAGGAGUG	478650_mir
	let-7f	CUAUACAAUCUAUUGCCUUCCC	77801_mir
		**Housekeeping**	
**miRNA**	Snord44	CCTGGATGATGATAAGCAAATGCTGACTGAACATGAAGGTCTTAATTAGCTCTAACTGAC	CSAAYWT Bach id:w1605853025000

Supplementary Table 6: Characteristics of tumor cell lines based on online databases

	SW837 (CCL235)	SW 480 (CCL228)	SW620 (CCL 227)
**Description**	Rectal adenocarcinoma (stage IV)	Colorectal adenocarcinoma (Duke's type B)	Rectal adenocarcinoma (Duke's type C)
**Tissue**	Human rectal	Human colon	Human colon but from a lymphatic metastasis
**Morphology**	Adhesive epithelial cell	Adhesive epithelial cell	Adhesive epithelial cell
**c-Myc mutation**	p.V92I; G->A*º^ Substitution - Missense	No*º	No*º
**p53 mutation**	p.R248W; G->A* p.R248W; C->Tº Substitution - Missense	p.R273H; c.818 G>Aº p.P309S; c.925 C>Tº Substitution - Missense	p.R273H; c.818 G>Aº p.P309S; c.925 C>Tº Substitution - Missense
**k-Ras mutation**	p.G12C; C-> A* p.G12C; G->Tº Substitution - Missense	p.G12V; C->A*º Substitution - Missense	p.G12V; C->A*º Substitution - Missense
**Apc mutation**	p.R213; C->T*^ p.R1450; C->T*º^ Substitution - Nonsense	p.Q1338; C->T*º Substitution - Nonsense	p.Q1338; C->T*º Substitution - Nonsense

* Cancer Cell Line Encyclopedia (CCLE), https://www.broadinstitute.org/ccle/home

º Catalog of Somatic Mutations in Cancer (COSMIC), http://cancer.sanger.ac.uk/cosmicous

^Heterozygous mutation

	SW837 (CCL235)	SW 480 (CCL228)	SW620 (CCL 227)
**c-Myc mutation**	V921I	Asp258Ser	Asp258Ser

We amplified genomic c-Myc DNA from cell lines using specific primers for each exon. PCR was performed in a 20 mL final volume reaction containing: 1 µL of DNA (100 µM), 2 µL of Buffer, 12.2 µL of water, 2 µL of Magnesium, 0.4 µL of dNTPs (10 mM each), 1 µL of each primer, and 0.2 µL of Taq polymerase under the following cycling conditions: 40 cycles of 94ºC for 30 s, 55ºC for 30 s and 72ºC for 30 s. PCR products were then analyzed by automatic sequencing (3700 Abi Prism).
