# Supplementary material for: Preoperative chemoradiotherapy for rectal cancer: the sensitizer role of the association between miR-375 and c-Myc
Source: Oncotarget. 2017 Jul 19;8(47):82294–302. doi: 10.18632/oncotarget.19393 (PMC5669890; doi:10.18632/oncotarget.19393)
Supplement: Supplementary file 2 [file oncotarget-08-82294-s002.docx]

**Supplementary Table 1.** Characteristics of the patients with rectal tumor

| **Nº sample** | **mRNA array** | **miRNA array** | **c-Myc**  **Taqman** | **miRNA**  **Taqman** | **miRNA-375** | **Normal Tissues** | **Sex** | **Age** | **CRT** | **cTN** | **STAGE_IN** | **SURG** | **TRG** | **DOWNST** | **DOWNS** | **RESP** | **CPR** |
| --- | --- | --- | --- | --- | --- | --- | --- | --- | --- | --- | --- | --- | --- | --- | --- | --- | --- |
| 1 | X | X | X | X | Low | NO | M | 63 | Capox | T4N1 | 3 | AR | 2 | 1 | 1 | Yes | No |
| 2 | X | X | X | X | High | NO | M | 71 | Cape | T3N0 | 2 | AR | 2 | 1 | 1 | Yes | No |
| 3 | X | X | X | X | High | NO | M | 77 | Cape | T3N1 | 3 | AR | 4 | 1 | 0 | No | No |
| 4 | X | X | X | X | High | NO | M | 67 | Cape | T3N0 | 2 | AR | 5 | 0 | 0 | No | No |
| 6 | X | X | X | X | High | NO | F | 83 | Cape | T3N0 | 2 | APR | 2 | 1 | 1 | Yes | No |
| 7 | X | X | X | X | High | NO | F | 63 | Cape | T3N2 | 4 | AR | 5 | 0 | 1 | No | No |
| 9 | X | X | X | X | Low | NO | M | 53 | Capox | T3N1 | 3 | AR | 1 | 1 | 1 | Yes | Yes |
| 11 | X | NO | NO | NO | NA | NO | M | 64 | Capox | T3N2 | 3 | AR | 2 | 0 | 1 | Yes | No |
| 12 | X | NO | NO | NO | NA | NO | M | 69 | Cape | T3N0 | 2 | AR | 3 | 1 | 1 | No | No |
| 13 | X | X | X | X | Low | NO | M | 69 | Cape | T3N0 | 2 | AR | 1 | 1 | 1 | Yes | Yes |
| 15 | X | X | X | X | High | NO | M | 71 | Cape | T3N0 | 2 | AR | 5 | 1 | 1 | No | No |
| 16 | X | X | X | X | High | NO | F | 62 | Cape | T3N1 | 3 | AR | 5 | 1 | 0 | No | No |
| 17 | X | X | X | X | Low | NO | F | 58 | Cape | T3N0 | 3 | AR | 1 | 1 | 1 | Yes | Yes |
| 18 | X | X | X | X | Low | NO | M | 50 | Capox | T4N0 | 2 | APR | 4 | 0 | 0 | No | No |
| 19 | X | X | NO | NO | NA | NO | M | 36 | Capox | T4N0 | 2 | AR | 5 | 0 | 0 | No | No |
| 20 | X | X | x | X | Low | NO | M | 54 | Cape | T3N0 | 2 | AR | 4 | 1 | 1 | No | No |
| 21 | X | NO | NO | NO | NA | NO | M | 47 | Capox | T3N0 | 2 | AR | 4 | 0 | 0 | No | No |
| 22 | X | X | X | X | High | NO | M | 45 | Capox | T3N0 | 2 | APR | 5 | 0 | 0 | No | No |
| 24 | X | X | X | X | High | NO | M | 47 | Capox | T3N1 | 3 | AR | 1 | 1 | 1 | Yes | Yes |
| 25 | X | X | NO | NO | NA | NO | M | 74 | Cape | T3N0 | 2 | AR | 4 | 1 | 1 | No | No |
| 27 | NO | NO | NO | NO | NA | NO | F | 61 | Cape | T3N1 | 3 | AR | 4 | 0 | 1 | No | No |
| 28 | NO | X | NA | NA | NA | NO | F | 37 | Capox | T3N2 | 3 | AR | 5 | 0 | 0 | No | No |
| 31 | NO | X | X | X | Low | NO | M | 54 | Cape | T3N0 | 2 | AR | 1 | 1 | 1 | Yes | Yes |
| 33 | NO | NO | NO | NO | NA | NO | M | 69 | Capox | T3N2 | 3 | APR | 3 | 0 | 0 | No | No |
| 34 | NO | X | X | X | High | NO | F | 70 | Cape | T3N2 | 3 | AR | 3 | 1 | 0 | No | No |
| 35 | NO | X | X | X | Low | NO | M | 61 | Capox | T3N1 | 3 | AR | 2 | 1 | 1 | Yes | No |
| 36 | NO | NO | NO | NO | NA | X | F | 76 | Cape | T3N0 | 2 | AR | 4 | 1 | 1 | No | No |
| 37 | NO | NO | NO | NO | NA | X | F | 64 | Cape | T3N2 | 3 | AR | 4 | 0 | 0 | No | No |
| 38 | NO | x | X | X | Low | X | M | 63 | Cape | T3N1 | 3 | AR | 2 | 1 | 1 | Yes | No |
| 39 | NO | NO | X | X | Low | X | F | 56 | Cape | T3N1 | 3 | AR | 3 | 1 | 0 | No | No |
| 40 | X | NO | NO | NO | NA | X | M | 62 | Cape | T3N1 | 3 | AR | 4 | 0 | 0 | No | No |
| 41 | NO | NO | X | X | Low | NO | M | 62 | Cape | T3N1 | 3 | AR | 1 | 1 | 1 | Yes | Yes |
| 42 | NO | NO | NO | NO | NA | X | M | 64 | Cape | T3N2 | 3 | AR | 3 | 0 | 0 | No | No |
| 43 | NO | NO | x | X | High | X | M | 56 | Cape | T3N1 | 3 | AR | 3 | 1 | 1 | No | No |
| 44 | NO | NO | NO | NO | NA | X | F | 52 | Cape | T3N1 | 3 | AR | 4 | 1 | 0 | No | No |
| 45 | NO | NO | X | X | High | NO | M | 62 | Cape | T3N1 | 3 | AR | 4 | 0 | 0 | No | No |
| 100 | NO | NO | X | X | High | NO | M | 69 | Cape | T3N2 | 3 | AR | 4 | 0 | 0 | No | No |
| 101 | NO | NO | NA | NA | NA | NO | M | 58 | Cape | T3N1 | 3 | AR | 2 | 1 | 1 | Yes | No |
| 102 | NO | NO | X | X | High | NO | M | 75 | Cape | T3N1 | 3 | AR | 2 | 1 | 1 | Yes | No |
| 107 | NO | NO | X | X | Low | NO | M | 66 | Cape | T3N0 | 3 | AR | 2 | 1 | 0 | Yes | No |
| 109 | NO | NO | X | X | High | NO | M | 69 | Cape | T3N1 | 3 | AR | 5 | 1 | 1 | No | No |
| 113 | NO | NO | X | X | Low | NO | M | 66 | Cape | T3N0 | 2 | AR | 4 | 0 | 0 | No | No |
| 116 | NO | NO | X | X | High | NO | F | 67 | Cape | T3N1 | 3 | AR | 2 | 1 | 1 | Yes | No |
| 117 | NO | NO | X | X | Low | NO | F | 50 | Cape | T3N1 | 3 | AR | 2 | 0 | 1 | No | No |
| 118 | NO | NO | X | X | Low | NO | M | 77 | Cape | T3N1 | 3 | AR | 5 | 0 | 0 | No | No |
| NA: data not available; AR: anterior resection; APR: abdominoperineal resection; Cap: Capecitabine; Capox: Capecitabine and Oxaliplatin; cTN: clinical stage; CRT: Chemoradiation; CRP: Complete pathologic response; DOWNS: downsizing; Downst: Downstaging; HART: Hartmann; LAR: Low anterior resection; miRNA-375: miR-375 expression levels measured by Taqman probes; Resp: response; Stage in: initial stage; Surg: surgical technique; TRG: Tumor Regression Grade | | | | | | | | | | | | | | | | | |
