# Supplementary material for: Preoperative chemoradiotherapy for rectal cancer: the sensitizer role of the association between miR-375 and c-Myc
Source: Oncotarget. 2017 Jul 19;8(47):82294–302. doi: 10.18632/oncotarget.19393 (PMC5669890; doi:10.18632/oncotarget.19393)
Supplement: Supplementary file 3 [file oncotarget-08-82294-s003.docx]

**Supplementary Table 2.** Patient characteristics stratified by response to treatment

| **mRNA microarrays (N=26)** | **Responders** | **Non-responders** | **p** |
| --- | --- | --- | --- |
| Sex |  |  | p>0.05 |
| Female | 5 (71%) | 2 (29%) |  |
| Male | 11 (58%) | 8 (42%) |  |
| Age (mean values ± standard deviation) | 62 | 59 | p>0.05 |
| Chemotherapy |  |  | p>0.05 |
| Capecitabine | 10 (67%) | 5 (33%) |  |
| Capecitabine + oxaliplatin | 6 (55%) | 5 (45%) |  |
| Surgical technique |  |  | p>0.05 |
| Low anterior resection | 13 (59%) | 9 (41%) |  |
| Abdominoperineal resection | 3 (75%) | 1 (25%) |  |
|  |  |  |  |
| **miRNA microarrays (N=22)** | **Responders** | **Non-responders** | **p** |
| Sex |  |  | p>0.05 |
| Female | 4 (67%) | 2 (33%) |  |
| Male | 8 (50%) | 8 (50%) |  |
| Age (mean values ± standard deviation) | 59 | 62 | p>0.05 |
| Chemotherapy |  |  | p>0.05 |
| Capecitabine | 8 (57%) | 6 (43%) |  |
| Capecitabine + oxaliplatin | 4 (50%) | 4 (40%) |  |
| Surgicaltechnique |  |  | p>0.05 |
| Low anterior resection | 10 (53%) | 9 (47%) |  |
| Abdomino perineal resection | 2 (66%) | 1 (33%) |  |
|  |  |  |  |
| **c-MycTaqman assay (N=31)** | **Responders** | **Non-responders** | **p** |
| Sex |  |  | p>0.05 |
| Female | 2(29%) | 5(71%) |  |
| Male | 12 (50%) | 12(50%) |  |
| Age (mean values ± standard deviation) | 63 | 62 | p>0.05 |
| Chemotherapy |  |  | p>0.05 |
| Capecitabine | 10 (40%) | 15 (60%) |  |
| Capecitabine + oxaliplatin | 4 (67%) | 2 (33%) |  |
| Surgicaltechnique |  |  | p>0.05 |
| Low anterior resection | 13 (48%) | 14 (52%) |  |
| Abdomino perineal resection | 1 (25%) | 3 (75%) |  |
|  |  |  |  |
| **miRNA Taqman assays (N=31)** | **Responders** | **Non-responders** | **p** |
| Sex |  |  | p>0.05 |
| Female | 2(29%) | 5 (71%) |  |
| Male | 12 (50%) | 12(50%) |  |
| Age (mean values ± standard deviation) | 63 | 61 | p>0.05 |
| Chemotherapy |  |  | p>0.05 |
| Capecitabine | 10(40%) | 15(60%) |  |
| Capecitabine + oxaliplatin | 4(67%) | 2(33%) |  |
| Surgical technique |  |  | p>0.05 |
| Low anterior resection | 13(48%) | 14 (52%) |  |
| Abdomino perineal resection | 1(25%) | 3(75%) |  |
