# Supplementary material for: Preoperative chemoradiotherapy for rectal cancer: the sensitizer role of the association between miR-375 and c-Myc
Source: Oncotarget. 2017 Jul 19;8(47):82294–302. doi: 10.18632/oncotarget.19393 (PMC5669890; doi:10.18632/oncotarget.19393)
Supplement: Supplementary file 4 [file oncotarget-08-82294-s004.docx]

**Supplementary Table 3.** 82 miRNAs manually annotated in database as related to c-Myc gene and/or identified in the literature. 29 candidate miRNAs described in more than one database or article are highlighted in grey

| **miRNA** | **Literature**^13-22^ | **mirTArBase** | **picTar** | **miRCancer** | **Targetscan** | **UCSC Genome browser** | **microRNA.org** | **miRSearch 3.0 (Exiqon)** |
| --- | --- | --- | --- | --- | --- | --- | --- | --- |
| miR-14 | X |  |  |  |  |  |  |  |
| miR-16 | X |  |  |  |  |  |  |  |
| miR-17-5p |  |  |  |  |  |  |  | X |
| miR-18a-5p |  | X |  | X |  |  |  |  |
| miR-20a-5p |  | X |  |  |  |  |  | X |
| miR-21 | X |  |  |  |  |  |  |  |
| miR-21-5p | X |  |  | X |  |  |  |  |
| miR-24-3p |  | X |  |  |  |  | X |  |
| miR-29b-2 | X |  |  |  |  |  |  |  |
| miR-30e | X | miR-30e-3p |  |  |  |  |  |  |
| miR-33 | X | miR-33a miR-33b |  |  |  |  |  |  |
| miR-33a-5p |  | X |  |  |  |  |  |  |
| miR-33b |  |  |  |  |  |  | X |  |
| miR-34a-5p |  | X |  | X |  |  | X | X |
| miR-34b-3p |  | X | X |  |  |  |  | X |
| miR-34b-5p |  |  | X |  |  |  |  | X |
| miR-34c-5p |  | X | X |  |  |  | X | X |
| miR-98-5p |  | X |  | X |  |  |  | X |
| miR-99a | X |  |  |  |  |  |  |  |
| miR-125 | X |  |  | X |  |  |  |  |
| miR-125a-3p | X | X |  |  |  |  |  |  |
| miR-125b | X |  |  |  |  |  |  |  |
| miR-126-5p |  | X |  |  |  |  |  | X |
| miR-127-3p | X |  |  |  |  |  |  |  |
| miR-135a-5p |  | X |  |  |  |  | X |  |
| miR-135b |  |  |  |  |  |  | X |  |
| miR-137 | X |  |  |  |  |  |  |  |
| miR-143 | X |  |  | X |  |  |  |  |
| miR-145 | X | X |  | X |  |  |  | X |
| miR-153 | X |  |  |  |  |  |  |  |
| miR-154 | X |  |  |  |  |  |  |  |
| miR-155-5p |  | X |  | X |  |  |  |  |
| miR-159c-3p | X |  |  |  |  |  |  |  |
| miR-183 |  |  |  |  |  |  | X |  |
| miR-186 |  |  |  |  |  |  | X |  |
| miR-188-5p | X |  |  |  |  |  |  |  |
| miR-1909 | X |  |  |  |  |  |  |  |
| miR-190b | X |  |  |  |  |  |  |  |
| miR-196b | X |  |  |  |  |  |  |  |
| miR-200a | X |  |  |  |  |  |  |  |
| miR-200c | X |  |  | X |  |  |  |  |
| miR-212-3p |  | X |  |  |  |  |  |  |
| miR-214 | X |  |  |  |  |  |  |  |
| miR-215 | X | X |  | X |  |  |  |  |
| miR-296 |  |  |  |  |  |  | X |  |
| miR-299-5p | X |  |  |  |  |  |  |  |
| miR-300 |  |  |  |  |  |  | X |  |
| miR-335-5p |  |  |  |  |  |  |  | X |
| miR-338-3p | X |  |  |  |  |  |  |  |
| miR-373-3p |  | X |  | X |  |  |  |  |
| miR-374a |  |  |  |  |  |  | X |  |
| miR-374b |  |  |  |  |  |  | X |  |
| miR-378 | X | miR-378a-3p |  |  |  |  |  |  |
| miR-381 |  |  |  |  |  |  | X |  |
| miR-409-3p | X |  |  |  |  |  |  |  |
| miR-429 |  | X |  | X |  |  |  | X |
| miR-449a |  |  |  |  |  |  | X |  |
| miR-449b |  |  |  |  |  |  | X |  |
| miR-450a | X |  |  |  |  |  |  |  |
| miR-450b-5p | X |  |  |  |  |  |  |  |
| miR-483-5p | X |  |  |  |  |  |  |  |
| miR-487b-3p |  | X |  |  |  |  |  |  |
| miR-494 |  |  |  |  |  |  | X |  |
| miR-494-3p |  | X |  |  |  |  | X |  |
| miR-539 |  |  |  |  |  |  | X |  |
| miR-561 | X |  |  |  |  |  |  |  |
| miR-590-5p | X |  |  |  |  |  |  |  |
| miR-599 |  |  |  |  |  |  | X |  |
| miR-622 | X |  |  |  |  |  |  |  |
| miR-630 | X |  |  |  |  |  |  |  |
| miR-671-5p | X |  |  |  |  |  |  |  |
| miR-720 | X |  |  |  |  |  |  |  |
| miR-744-5p |  | X |  |  |  |  |  |  |
| miR-765 | X |  |  |  |  |  |  |  |
| miR-1183 | X |  |  |  |  |  |  |  |
| miR-1224-5p | X |  |  |  |  |  |  |  |
| miR-1471 | X |  |  |  |  |  |  |  |
| miR-let7a-5p |  | X |  |  |  |  |  | X |
| miR-let7c-5p |  | X | X | X |  |  |  | X |
| miR-let7e | X |  |  | X |  |  |  |  |
| miR-let-7f-5p |  | X |  |  |  |  |  | X |
| miR-let7g-5p |  | X |  |  |  |  |  | X |
